# ASPA: Advanced Strong Pseudonym based Authentication in Intelligent Transport System

**DOI:** 10.1371/journal.pone.0221213

**Published:** 2019-08-22

**Authors:** Qazi Ejaz Ali, Naveed Ahmad, Abdul Haseeb Malik, Waheed Ur Rehman, Aziz Ud Din, Gauhar Ali

**Affiliations:** 1 Department of Computer Science, University of Peshawar, Peshawar, Pakistan; 2 Shaikh Zayed Islamic Centre, University of Peshawar, Peshawar, Pakistan; Wuhan University, CHINA

## Abstract

Intelligent Transport System (ITS) uses the IEEE 802.11P standard for the wireless communication among vehicles. A wireless ad hoc network of vehicles is established to improve road safety, comfort, security, and traffic efficiency. Wireless communication in ITS leads to many security and privacy challenges. Security and privacy of ITS are important issues that demand incorporation of confidentiality, privacy, authentication, integrity, non-repudiation, and restrictive obscurity. In order to ensure the privacy of vehicles during communication, it is required that the real identity of vehicles should not be revealed. There must be robust and efficient security and privacy mechanisms for the establishment of a reliable and trustworthy network. Therefore, we propose Advanced Strong Pseudonym based Authentication (ASPA), which is a distributed framework to handle the security and privacy issues of vehicle communications in ITS. ASPA only allows vehicles with valid pseudonyms to communicate in ITS. Pseudonyms are assigned to vehicles in a secure manner. The pseudonym mappings of vehicles are stored at different locations to avoid any chance of vehicle pseudonyms certificates linkability. In addition, the most recent communication pseudonyms of a malicious vehicle are revoked and are stored in the Certificate Revocation List (CRL) that results in small size of the CRL. Therefore, the CRL size does not increase exponentially. The distributed framework of ASPA guarantees, the vehicles privacy preservation in the real identities mapping and revocation phase. The empirical results prove that ASPA is robust and efficient with low computational cost, overhead ratio, average latency, and an increased delivery ratio.

## I. Introduction

Intelligent Transport System (ITS) is one of the derived forms of Information and Communication Technology (ICT) that is established on vehicular communication. ITS enabled vehicles allow ITS users to obtain updated information of traffic situations. ITS reduces the cost of fuel in traveling and results in efficient driving [1–3]. Deficiency in driving seriousness and population growth results in un-necessary delays, congestions, and accidents in journeys [1,4]. Delays in traveling, road accidents, and congestions can be reduced through ITS [5].

**Fig 1:**
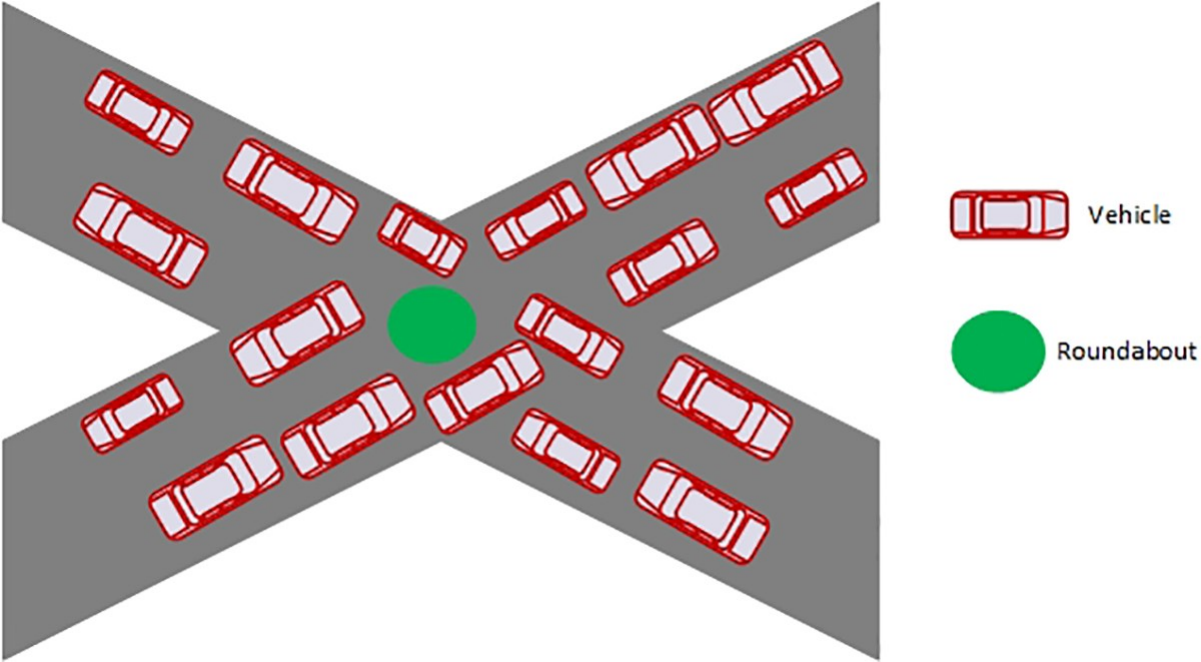
Scenario without ITS.

Vehicular Ad hoc Networks (VANETs) is an important part of ITS [1,4]. VANETs use the ITS architecture to reduce road accidents and it provides reliable safety messages known as beacons, which includes information of vehicle’s positions, headings, speeds, and traffic situations [1]. One of the scenarios of non-seriousness of drivers is shown in Fig 1. In order to provide better services to society, there is a need to incorporate intelligence into the transport system.

**Fig 2:**
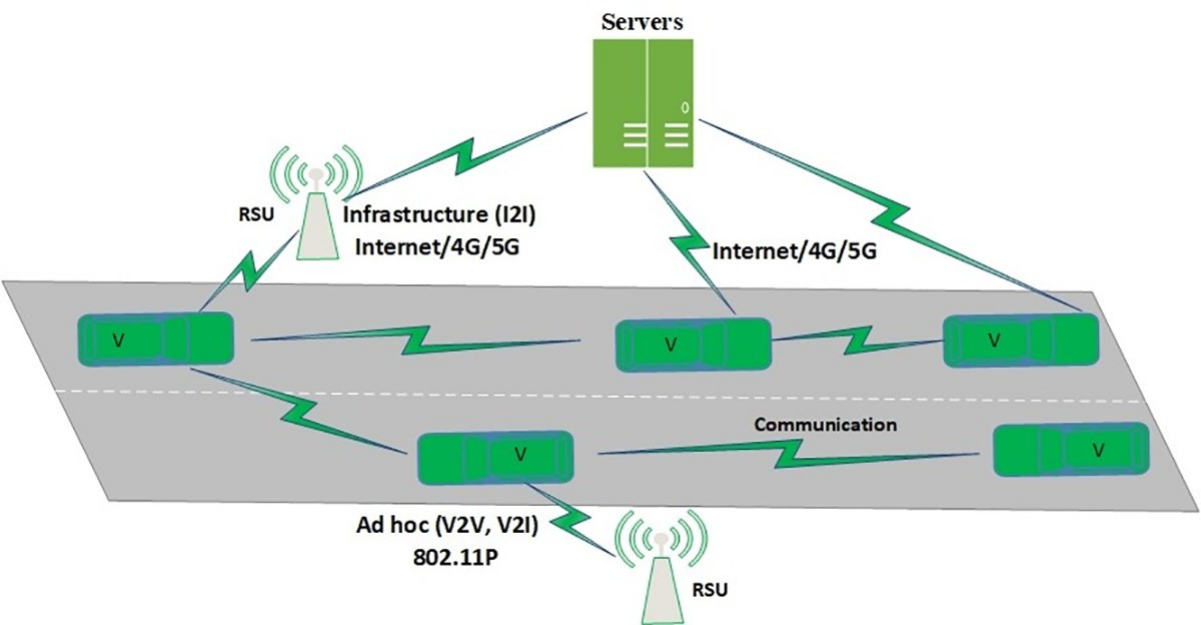
ITS scenario.

An ITS consists of Intelligent Transport System-Stations (ITS-Ss), which can be either Road Side Units (RSUs), vehicles, and servers [1]. Each vehicle in ITS is equipped with an On Board Unit (OBU) that enables it to participate in ITS communication. One of the ITS scenarios is shown in Fig 2.

Generally, ITS applications can be categorized into Advanced Traffic Management Systems (ATMS), Advanced Driver Assistance Systems (ADAS), and Advanced Traveler Information Systems (ATIS) [1,6]. ATMS, ADAS, and ATIS applications are achieved through Cooperative Awareness Messages (CAMs) [7,8]. CAMs are known as Basic Safety Messages (BSMs) in the United States [7,9]. BSMs include slow vehicle indications, speed control, collision warnings, reverse parking assistance, intersection collision warnings, hazardous locations, visibility warnings, wind, and road work messages [7,8].

The main applications of ITS are focused on traffic efficiency and road safety of vehicles. Other applications may include infotainment applications such as public transport information, internet booking, trip reservation, trip matching services, route planning, local electronic commerce, media downloading, real time traffic status, and financial services.

In order to provide V2X communication, which includes both Vehicle to Vehicle (V2V) and Vehicle to Infrastructure (V2I), the IEEE 802.11P standard is used [7,10,11]. This standard is considered as Dedicated Short Range Communication (DSRC) or Wireless Access in Vehicular Environment (WAVE) [1,7]. In ITS, due to the ad hoc and wireless environment, security and privacy issues are introduced. Security and privacy problems in ITS can jeopardize the privacy of a vehicle. An attacker can use false messages to misguide ITS users or collect vehicles confidential data or track vehicles [1,12]. To protect the privacy of vehicles from different attackers, reliable and efficient security and privacy approaches are required. The IEEE 1609.2 standard addresses the security issues in ITS [13]. This standard advises that a Certification Authority (CA) should issue digital certificates to vehicles in ITS. The digital certificates of a vehicle should be revoked if any malicious activity is detected. The malicious vehicle should be scrubbed from ITS. Applications, which use BSMs for communication, require conditional anonymity [14]. Unfair use of ITS by malicious vehicles should be controlled to provide privacy and secure communication.

In ITS, vehicles broadcast safety messages periodically, to update other ITS-Ss regarding their present status (e.g. velocity, position, and direction). This information is very sensitive and can be misused if leaked [15]. In ITS, to furnish secure and reliable communication, the classic security features should be kept. The classic security features are authentication, non-repudiation, privacy, and integrity [16,17]. Pseudonyms can be utilized to preserve the real identity of vehicles [16,18]. However, to support privacy, pseudonyms should not be easily linkable to the real identity of a vehicle. In order to provide this un-linkability feature, pseudonyms are required to change at regular intervals [19].

Pseudonym based approaches addressed in [20,21] exercise simple cryptography to preserve the real identity of vehicles. However, these approaches incur high communication and computational overheads. In ITS, onion routing based approaches are also not viable solutions, because of the high computational and communication overheads [22]. Schaub et al. [23] discussed that due to Sybil attacks, autonomous pseudonyms should not be used. A pseudonym to real identity information may be integrated into certificates [24]. However, this direct linkability of pseudonym to real identity in certificates can jeopardize the source vehicles privacy.

Wang et al. [25] suggested the use of two servers. One of the servers issues pseudonyms and the other server checks the reputation of vehicles. However, in order to examine the reputation of vehicles produces communication delay. This delay can cause incorrect information dissemination. Rajput et al. [26] addressed the use of primary and secondary pseudonyms. The CA provides primary pseudonyms, while secondary pseudonyms are provided by RSUs. However, RSUs are deployed in an open infrastructure and are prone to side channel attacks [27].

Whitefield et al. [28] discussed that vehicles after malicious activities detection should be revoked from VANETs. In ITS, only minimum information of a vehicle can be exposed to other ITS-Ss i.e. vehicles and service providers [21]. Whitefield et al. [28] discussed that there should be conditional anonymity in ITS, only in case of an awful activity, there should be revocation of a malicious vehicle. In ITS, malicious vehicles should be banned otherwise honest vehicles can be misguided. In order to achieve useful security and privacy, more than one beacons relevant to one vehicle should not be joined, otherwise, semantic linking and syntactic linking attacks are possible [29].

Therefore, in ITS, after considering the aforesaid issues, there is the necessity of efficient and scalable security and privacy schemes. These schemes should allow only authorized vehicles to take part in ITS network and should preserve the real identities of authorized vehicles in communication. Advanced Strong Pseudonym based Authentication (ASPA) in ITS is an improved form of our preliminary contribution [7]. In this paper, the proposed framework is designed to be more robust and scalable by further reducing the computational costs.

The contributions of this paper are as follows:

- A novel framework is proposed to involve multiple authorities for pseudonyms formation.
- The single authoritative behavior of the certificate authority is eliminated through distributed trust management methodology.
- The linkability of pseudonyms mapping at a single authority level is eliminated.
- A novel conditional revocation scheme is proposed in which upon malicious/awful activity only, a malicious vehicle is revoked through distributed mapping.
- The proposed framework is implemented using different security techniques.
- To examine the usefulness, appropriateness, and robustness of the proposed framework, it is analyzed through pervasive simulations and security analysis.

The rest of the paper is structured as: Related work is presented in Section II. The preliminaries of the proposed framework are discussed in Section III. The proposed revocation process is discussed in Section IV. In Section V performance analysis is discussed. Security analysis is presented in Section VI. In Section VII conclusion and future work is presented.

## II. Related work

Intermittent communication in an ITS network requires reliable verification of the authenticity and integrity of safety messages or beacons [30]. Researchers have been actively working in ITS to preserve the real identities of vehicles. However, still, there is a big challenge for researchers to develop efficient and scalable security and privacy schemes. Generally, in ITS, privacy protection approaches are classified into Pseudonym Based (PB) schemes and Ring Signature Based (RSB)/Group Signature Based (GSB) approaches.

In most PB schemes, asymmetric/public key cryptography is used. In these schemes, the message is signed through the private key, while the signature is verified through the corresponding public key. Generally, in these approaches, a CA issues certificates along with pseudonyms and the mapping between the pseudonym and the real identity is performed by the CA.

Raya et al. [16] suggested a bulk of pseudonyms generation and its distribution to the vehicles. The source vehicle randomly selects a pseudonym from the bulk and signs the message through its private key, the receiving vehicle verifies the authenticity of the messages through the corresponding public key certificate. In case of a malicious activity, the CA maps the real identity of the vehicle. However, CA is a single threat model having all mapping information of vehicles. The authors of [31] presented a scheme that provides bulk of pseudonyms certificates to the vehicle. However, in this scheme the storage overhead is high. Similarly, in order to revoke bulk of pseudonyms, CRL size grows exponentially. Therefore, to reduce the size of CRL, hash chain idea is suggested by Sun et al. [32]. However, computation of hash chains incurs an additional computational cost.

Calandriello et al. [33] presented a scheme in which a common key pair is provided to vehicles that can be compromised. In addition, each time it is verified that the message is from a revoked vehicle or not. This produces an extra overhead. Rajput et al. [34] presented an approach in which RSUs take part in pseudonym generation and is prone to side channel attacks. Boneh et al. [35] and Zhang et al. [36] presented identity based verification schemes. In these approaches, tamper proof devices are used for pseudonym based identity certificates generation and storage. However, these schemes are prone to Sybil and colluding attacks. Lue et al. [37] discussed conditional privacy preserving protocol that allows RSUs to provide short time pseudonym keys to vehicles. However, RSU can be attacked easily due to its nature of deployment. Singh et al. [38] presented a scheme for beacons verification, anonymous credentials and camenish lysyanskaya signature is used. However, in this scheme, the computational and communication overheads are high.

Lefevre et al. [39] proposed an approach that allows direct linkability between the pseudonym and real identity of a vehicle. However, this linkability can jeopardize the privacy of vehicles. Schaub et al. [24] suggested a scheme in which the Registration Authority (RA) is responsible for the mapping of a pseudonym and is a single point of attack. Alheeti et al. [40] presented an approach that can prevent only external attacks. However, this scheme is vulnerable to internal attacks.

Kamat et al. [41] suggested the idea of a Trusted Authority (TA), which issues pseudonym certificates to vehicles. In this scheme, TA is a single threat model, because TA is responsible for pseudonym certificates generation and revocation. In addition, revocation information of vehicles are stored on base stations that are positioned in open areas and can easily be targeted. Wang et al. [42] discussed an approach that allows Key Management Centre (KMC) to keep all the vehicles information. KMC is a single threat model because it contains all the relevant information of vehicles. Kumar et al. [43] presented a pseudonym scheme, however the scheme provides low privacy.

TSO et al. [44] presented the idea of Certificate Less -Public Key Cryptography (CL-PKC) scheme to reduce the signature generation computational overhead and storage requirements. However, this scheme lacks support for revocation of malicious vehicles and is prone to active and passive attacks. Horng et al. [45] discussed an approach for V2I communication but lacks support for revocation of malicious vehicles. In addition, the signature authentication process can be performed by RSUs. However, RSUs are located in open infrastructure and are prone to side channel attacks [46].

In RSB/GSB schemes [47–49], vehicles group are formed and the public key certificates are used to check the authenticity of vehicles in a group. The group keys are used to hide the real identity of a vehicle in a group from other members of the group. In RSB/GSB schemes, the messages for a group are signed through a respective ring/group key. However, there is a limitation of scalability in RSB/GSB approaches. Shamir et al. [48] presented an early scheme of GSB in which RSU is used to sign and authenticate messages. However, due to side channel attacks, RSUs cannot be allowed to actively participate in ITS communication.

Zhang et al. [49] discussed a scheme to manage a group in which RSU acts as a group manager. However, due to its nature of deployment RSUs can be compromised. Liu et al. [50] presented a revocable ring signature scheme to secure ITS. However, this scheme is not scalable because it is for a particular ring/group. The work proposed in [51] suggested for vehicles security and privacy, revocable ring signature. However, this approach incurs high overhead due to the timely distribution of CRL among all vehicles, as the CRL size is increasing exponentially. Zhu et al. [52] presented a GSB approach, however, the scheme is not scalable. Hu et al. [53] proposed a hybrid approach of security but is prone to side channel attacks [1].

In GSB schemes there are issues of scalability, group management, pairing based computational costs, and full trust on the group manager. Similarly, most PB approaches suffer from high computational costs, communication overheads, security threats, and storage requirements, due to large size of CRL and bulk of pseudonyms in the vehicle OBU. Related work shows that reliable and efficient trustworthy schemes are still a big challenge for the researchers. In this paper, the next section presents a new framework, Advanced Strong Pseudonym based Authentication (ASPA), to generate pseudonyms in a distributed manner with a higher degree of secure communication among vehicles and service providers. In the proposed framework, vehicles privacy is addressed efficiently.

## III. Preliminaries

This section consists of the proposed ASPA framework, assumptions, design objectives, security tools, privacy metrics, the ASPA protocol, and the attack model.

### A. ASPA framework

Secure communication in ITS requires the protection of actual identities of vehicles. In the ASPA framework, the real identities of vehicles cannot be revealed by a single authority. In addition, in the case of an awful behavior, malicious vehicles should be revoked and accountability should be performed. In order to avoid linkability, the ASPA framework is implemented in a distributed manner to use fictitious identities and certificates. The ASPA framework consists of:

- **Vehicular Manufacturing Company (VMC):** An initial pseudonym is provided by the VMC to the vehicle in a secure link. In order to limit the single authoritative behavior of CA, the ASPA framework considers the manufacturing industry. In the ASPA framework, the real identity of a vehicle is hidden from the CA. In the proposed framework the vehicle interaction is considered only once with the VMC or if ownership of the vehicle is changed.
- **Certification Authority (CA):** After successful verification of the vehicle from the VMC, the CA issues Long Term Certificate (LTC) to the vehicle in a secure channel. The expiration time of a vehicle LTC in a normal situation is one year or the CA can set it in the field of the timestamp. Therefore, the vehicle can interact with the CA for the LTC after every year or as given in the timestamp field.
- **Long Term Certification Authority (LTCA):** After a trustworthy authentication process, the LTCA issues a Pseudonym Certificate (PC) in a secure channel to the vehicle. The expiration time of a vehicle PC in a normal situation is six months or the LTCA can set it in the field of timestamp but must be less than the LTC lifetime. Therefore, the vehicle can interact with LTCA for the PC after every six months or as given in the timestamp field.
- **Pseudonym Provider (PP):** The Short term Communication Pseudonyms (SPCs) are provided by the PP or cascaded PPs in a secure channel to the vehicle. This is done after a trustworthy authentication process. In order to get SPCs for V2V communication, the interaction of the vehicle with PP is frequent.
- **Source vehicle:** The safety messages/beacons originator (*Vi*), uses its private key to sign the safety messages and disseminate them. The SPC and the corresponding public key are appended with the sign beacons.
- **Receiving vehicle:** The receiving vehicle (*Vj*) verifies the beacons/safety messages through the SPC. The verification of the signature is performed through the corresponding public key. In case of spurious beacons, the *Vi* is reported for revocation from ITS to PP, CA, and Law Enforcement Organization (LEO). The *Vj* discards a beacon, if a beacon signature is not verified.

In the proposed framework of ASPA, the SPCs validity is between 10 to 50 milliseconds. The SPCs validity lifetime is kept small to ensure un-linkability of communication pseudonyms. In case, if a vehicle is detected awful, no more SPCs can be issued to the vehicle. Furthermore, all the previously issued SPCs should be isolated from ITS network. The LEO can reveal the real identity of a vehicle only after detection of an awful activity. In case, if the vehicle ownership changes, all the issued certificates should be revoked. This revocation should provide inaccessibility of the previous private communication and real identity protection. The new owner requires the repetition of steps from VMC to PP as discussed in Section III-F.

### B. Assumptions

It is inferenced that the real identity of a vehicle is disclosed by the VMC to LEO once a vehicle is found malicious. All the aforementioned entities should have secure and trustworthy communication. A PP will be detached, if it is compromised. In the ASPA framework for V2X communications, RSUs act as routers. RSUs do not actively participate in the generation of communication pseudonyms. This is because of side channel attacks. A vehicle can request for pseudonyms from the authorities directly using 4G/5G/Internet or through RSUs. In order to provide un-linkability of SPCs by the attacker, there will be a number of PPs. All the functional entities in the proposed ASPA framework, clocks are synchronized. This synchronization is required because of timestamps in the secure communication.

### C. Design objectives

The design objectives of the proposed ASPA framework are as follows:

- **Reduced computational cost:** The computational cost of the proposed framework will be reduced, to efficiently work in more complex scenarios. Therefore, the ASPA becomes more robust and scalable.
- **Confidentiality and authentication:** The communication between vehicles and all the service providers will be encrypted. Similarly, without disclosure of the true identity of a legitimate vehicle, it will be verified and authorized. The receiving vehicle will authenticate a source vehicle and its beacons without disclosure of its valid identity.
- **Integrity of communication:** If beacons are altered, the beacons signature will not be verified. Therefore, unproven beacons will be shredded and discarded.
- **Non-repudiation:** If a signature is verified, this will show the authenticity of source vehicle beacon. In this case, the communication cannot be refused.
- **Revocation:** If a vehicle or a pseudonym is revoked, again it will not be used in the ITS.
- **Restrictive obscurity:** Restrictive obscurity is rendering in the ASPA framework. The privacy of a vehicle will be preserved if it follows the ASPA rules. Only in case of an awful activity, the real identity of a vehicle will be revealed/disclosed.

### D. Security primitives

ASPA implements a sequence of secret and public key cryptographic strategies. Secret Key Cryptography (SKC) processes are more efficient than Public or Asymmetric Key Cryptography (AKC) processes. However, the non-repudiation service cannot be provided only through SKC. Therefore, to address security and privacy features efficiently, we merge the SKC and AKC strategies. In ASPA framework, for SKC, we implement Advanced Encryption Standard (AES) and for AKC, two schemes are implemented. One of the AKC schemes is Rivest, Shamir, and Adleman (RSA), while the other scheme is the Digital Signature Algorithm (DSA).

A key pair of private and public keys are generated through the vehicle OBU. The signature is generated through the private key, the corresponding public key is transmitted along with beacons to verify the authenticity of beacons at the receiving vehicle. The following two methods are considered to generate the key pairs, which are as follows:

### 1. Method 1

- The generation of two random prime numbers is performed. For instance, *a* and *b* are generated, *n* is calculated, such that:

n=(a)(b). (1)

- The computation of public key (*pb*) is performed through Eq (2). Where, Greatest Common Divisor (GCD) between *pb* and totient function (*φ*(*n*)) is 1.

GCD(pb,φ(n)), (2)

where,

φ(n)=(a−1)(b−1). (3)

- The computation of private key (*pr*) is performed through Eq (4).

(pb)(pr)≡1mod(φ(n)). (4)

Where, the congruence property is satisfied by using Eq (5).

((pb)(pr))−1)modφ(n)=0. (5)

Therefore, private key is {*pr*} and public key is {*pb*}.

### 2. Method 2

- Generate a prime number of size 2*X*, where *X* = 128 bits.
- Generate a number *b* such that:

GCD(b,a)=1. (6)

- Calculate *c*, such that:

$$c=d^{\frac{\varphi (n)}{b}},$$ (7)

where,

φ(n)=a−1, (8)

such that:

d<φ(n). (9)

Similarly,

$$d^{\frac{\varphi (n)}{b}}(mod(a))>1.$$ (10)

- Generate a private key such that:

pr<b. (11)

- Calculate public key such that:

$$pb=c^{pr}(mod(a)).$$ (12)

Therefore, private key is {*pr*} and public key is {*pb*}.

**Fig 3:**
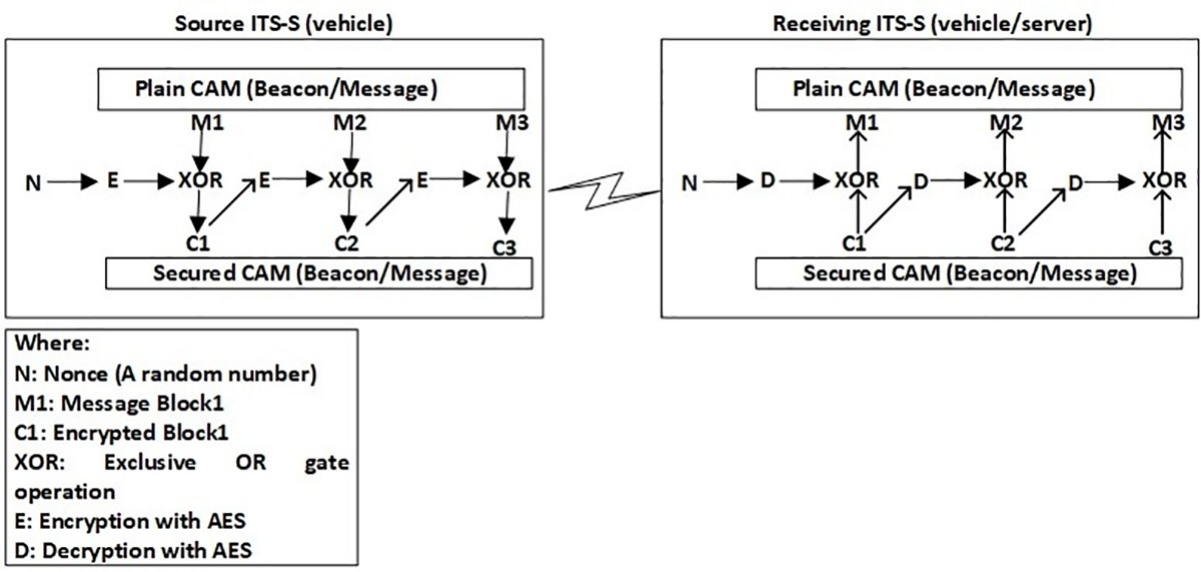
ASPA, CFM operation.

In the proposed ASPA framework, AES uses 128 bits (16 bytes) data block and secret key size is 128 bits (16 bytes). In case, if the safety message size is more than 16 bytes, the Cipher Feedback Mode (CFM) scheme is implemented [54]. In case of smaller size of a data block from 16 bytes, padding is considered to make the size of data block compatible with the key size. For the first block of data, a random number known as a nonce (*N*) is exclusive OR (XOR) after encryption process. Similarly, the previous block of ciphertext acts as a random number for the next block of plain text. Fig 3, shows the ASPA, CFM process. The message will be authenticated, after an ITS-S (vehicle or server) gets the secured message.

### E. Privacy metrics

A trustworthy privacy scheme should guarantee a high level of obscurity. A range of metrics are discussed, to assess the level of privacy through pseudonyms. The metrics that will be used for evaluation are as following:

- **Anonymity set size:** The size of Anonymity Set (AS) is the number of the vehicles that are included in the AS [55]. In security and privacy schemes, the AS size should be larger than one. However, the AS metric assumes the entire range of vehicles is adequately being the victim. Therefore, as discussed in [56], the AS metric cannot be examined to express that the attacker, targeted how many vehicles in the network. Therefore, preferably of AS, entropy is suggested [56].
- **Entropy of the AS size:** Information theory provides the concept of entropy. Entropy describes anxiety in a random variable. The number of vehicles are shown by a random variable. For instance, the probability of a random variable *N* is as follows:

$$y_{j}=p_{rob}(N=j).$$ (13)

Where, *j* in Eq (13) shows a possible range of vehicles, which can be viewed by *N*, with probability *y*_*j*_*>0*. The probability *y*_*j*_ shows the contents of the messages that can be associated with the vehicles. Therefore, the entropy can be measured through Eq (14).

$$H(N)=-\sum_{j=1}^{\left|AS\right|}y_{j}log_{2}(y_{j}).$$ (14)

In Eq (14), *y*_*j*_ shows a vehicle probability, while *j* represents the attacked vehicles. If all vehicles have the same attack probability, the AS has a uniform distribution of probabilities. The entropy maximum value can be achieved by Eq (15).

$$\forall_{j}:y_{j}=\frac{1}{\left|AS\right|},\;H_{\max}=-\sum_{j=1}^{\left|AS\right|}y_{j}\log_{2}(y_{j})=\log_{2}|AS|.$$ (15)

For instance, in an ITS, if the number of the vehicles is 25 and we inference that there is an equal probability for all vehicles to be attacked, then *y*_*j*_ = 1/25, *y*_*j*_ = 0.04 and 4.64 is the entropy. A greater AS size is achieved through a high value of entropy. In ITS, as the vehicles are increasing, there will be an increase in the entropy.

- **Anonymity level:** If there is no past information of vehicles AS with an attacker, the following difference can be used to describe the attacked data: (*H*_*max*_−*H*(*N*)). Where *H(N)* is the sufficient AS size and the ultimate entropy is *H*_*max*_. The degree of entropy i.e., d is suggested by Diaz [14] that is a normalized amount in [0, 1] range. Therefore, Eq (16) is used to calculate the degree of anonymity.

$$d=1-\frac{H_{max}-H(N)}{H_{max}}=\frac{H(N)}{H_{max}}.$$ (16)

The proposed ASPA framework tries to address a high level of anonymity through a robust and distributed mechanism.

### F. ASPA proposed protocol

**Fig 4:**
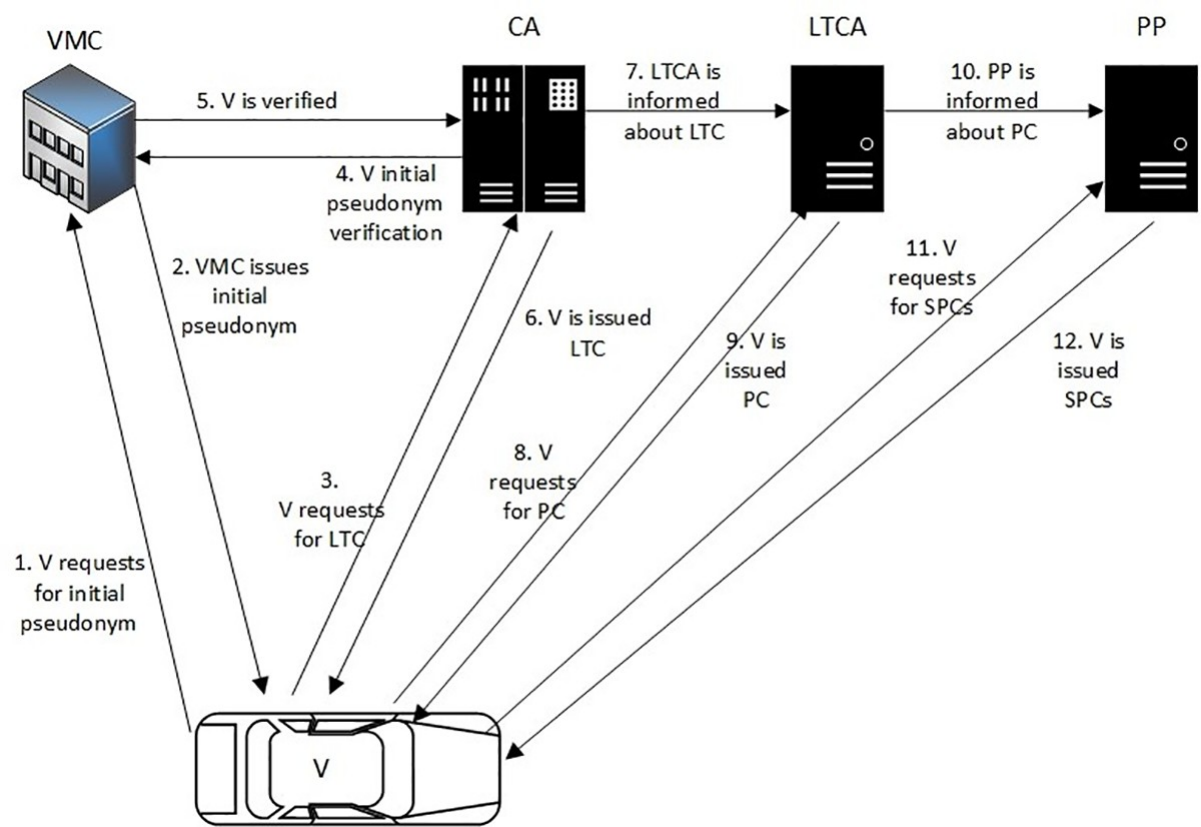
ASPA framework.

The VMC pre-loads an ITS-S (vehicle) with a secret key. The vehicle requests through the secret key from the VMC for an initial pseudonym. Furthermore, the vehicle requests for LTC from CA. The credentials of the vehicle are checked by the CA in CRL. If the vehicle does not exist in the CRL, Algorithm 1 is executed. The notations used in the ASPA protocol are given in Table 1, while Fig 4 shows the working process of ASPA framework.

**Table 1:** ASPA notations.

Notations	Description
V	ITS-S (vehicle)
PP	Short Time Pseudonym Provider for vehicular communication
S_k_	Session key
K_VVMC_	Secret key shared by V and VMC
V_i_	Source vehicle
V_j_	Receiving/affected vehicle
P_1_	Pseudonym 1
P_2_	Pseudonym 2
P_3_	Pseudonym 3
Pk_LTCA_	Public key of LTCA
Sk_1_	Session key for V and LTCA
Sk_2_	Session key for V and PP
K_v_	Secret session key for CA and V
Pk_VMC_	Public key of VMC
Pk_CA_	Public key of CA
Pk_PP_	Public key of PP
LT	Life Time of pseudonym
TS	Time Stamp
||	Concatenation
N	Nonce a random number
Token	Only for the authorized vehicle/server
K_LTCA_	Secret key shared by CA and LTCA
K_PP_	Secret key shared by LTCA and PP
K_Vi_	Secret key of V_i_
Pk_Vi_	Public key of V_i_
Ms	Milliseconds
KB	Kilobytes
MB	Megabytes
RA	RSA and AES
DA	DSA and AES
RD	RSA and DSA
/	Or

Algorithm 1 ASPA protocol

1:*V*→*VMC*:*K*_*VVMC*_[*ID*_*VVMC*_||*N*||*ID*_*V*_]

2:*VMC*→*V*:*K*_*VVMC*_[*P*_1_||*ID*_*VMC*_||*ID*_*CA*_||*N*||*K*_*V*_]

3:*V*→*CA*:*Pk*_*CA*_[*P*_1_||*ID*_*VMC*_||*K*_*V*_]

4:*CA*→*VMC*:*Pk*_*VMC*_[*P*_1_||*ID*_*VMC*_||*K*_*V*_]

5:*VMC*→*CA*:*Pk*_*CA*_[*Ok or decline*] *if ok then*

6:*CA*→*V*:*K*_*V*_[*Sk*_1_||*P*_2_||*TS*_1_||*LT*_1_||*ID*_*LTCA*_||*Token*_*LTCA*_]

7:*CA*→*LTCA*:*Pk*_*LTCA*_[*P*_2_||*SK*_1_||*TS*_1_||*LT*_1_||*ID*_*LTCA*_] *or Token*_*LTCA*_

8:*V*→*LTCA*:*Sk*_1_[*P*_2_||*ID*_*LTCA*_||*Token*_*LTCA*_] *Where*,          *Token*_*LTCA*_:*K*_*LTCA*_[*P*_2_||*ID*_*LTCA*_||*TS*_1_||*LT*_1_]

9:*LTCA*→*V*:*Sk*_1_[*P*_3_||*Sk*_2_||*LT*_2_||*TS*_2_||*Token*_*PP*_||*ID*_*PP*_]

10:*LTCA*→*PP*:*Pk*_*PP*_[*P*_3_||*Sk*_2_||*ID*_*PP*_||*TS*_2_||*LT*_2_] *or Token*_*PP*_

11:*V*→*PP*:*Sk*_2_[*P*_3_||*ID*_*PP*_||*Token*_*PP*_] *Where*, *Token*_*PP*_:*K*_*PP*_[*P*_3_||*ID*_*PP*_||*TS*_2_||*LT*_2_]

12:*PP*→*V*:*Sk*_2_[*P*_4_||*P*_5_||*P*_6_||*P*_7_||*TS*_3_||*LT*_3_]

The proposed ASPA protocol elaborates that:

- Step 1: The request of the vehicle from the VMC is performed through K_VVMC_ for an initial pseudonym.
- Step 2: The vehicle gets an initial pseudonym through KVVMC from the VMC.
- Step 3: It shows the request of the vehicle for the LTC from the CA through Pk_CA_.
- Step 4: The authentication of the vehicle is performed by the CA from the VMC through Pk_VMC_.
- Step 5: The vehicle is verified or declined by the VMC through Pk_CA_.
- Step 6: After the vehicle is successfully verified from the VMC, the CA issues LTC to the vehicle through KV. If the vehicle is found malicious, the CA reports it to LEO for accountability.
- Step 7: The LTCA is informed by the CA through Pk_LTCA_ about the LTC.
- Step 8: It shows the request of the vehicle for PC from the LTCA through Sk_1_. The LTCA checks both the tokens that are forwarded by the vehicle and the CA. If the tokens are verified, then Step 9 is executed.
- Step 9: The vehicle gets a PC from the LTCA through Sk_1_.
- Step 10: PP or cascaded PPs are informed by the LTCA regarding the PC of the vehicle in a secure link.
- Step 11: It shows the request of the vehicle for SPCs from PP through Sk_2_. This request is based on the PC that is issued by the LTCA.
- Step 12: The PP verifies the request of the vehicle and issues SPCs through Sk_2_ for V2X communication.

**Fig 5:**
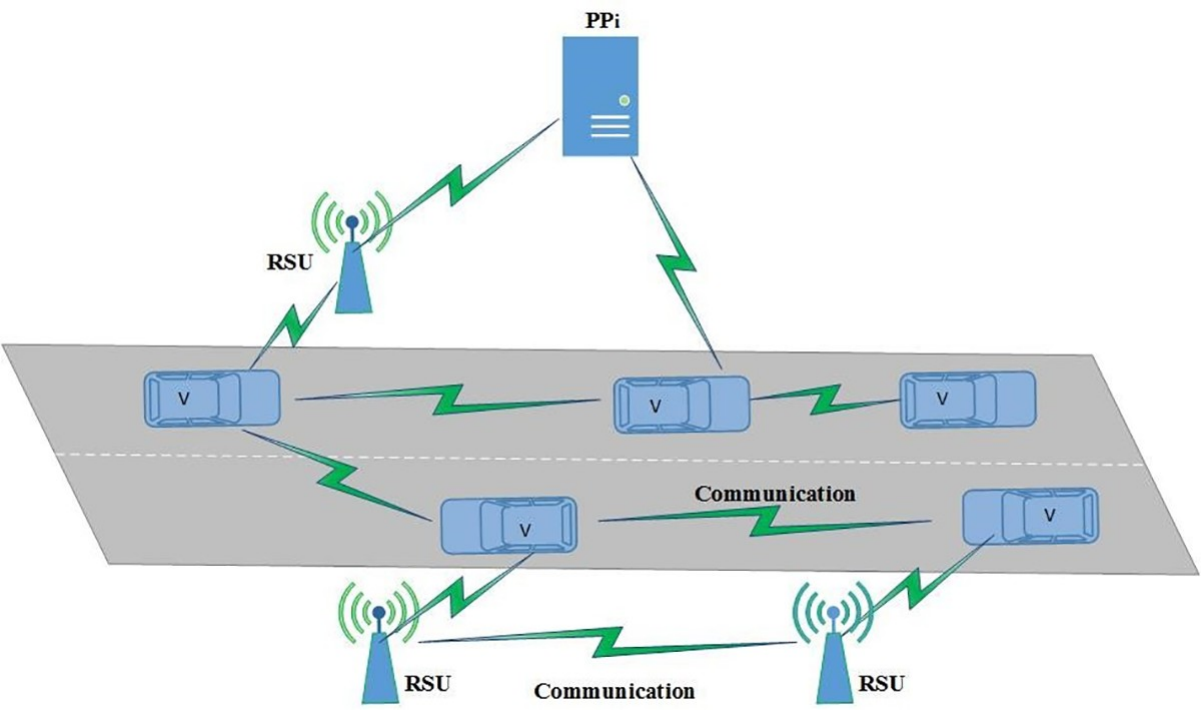
ASPA communication scenario.

The vehicle registration process pseudo code is discussed in Algorithm 2. Once PP or cascaded PPs issue, SPCs to the vehicle, the vehicle communicates through SPCs with other vehicles and RSUs as shown in Fig 5. If a bogus beacon is received from a *V*_i_, *V*_*j*_ reports LEO regarding *Vi* revocation. The revocation process of a malicious vehicle is discussed in Section IV.

Algorithm 2 Pseudo code of ASPA vehicle registration

1: if *V* requests CA

2:    *V* is cross checked with VMC

3:    *V* is authorized by VMC

4: end if

5: if *V* is authenticated

6:    CA issues LTC to *V*

7:    *V* requests LTCA for PC

8: end if

9: if *V* is authenticated

10:    LTCA issues PC

11:    *V* requests PP for SPCs

12: end if

13: if *V* is authenticated

14:    PP issues SPCs for communiation

15: end if

### G. Attack model

In the attack model of ASPA framework, different threats are considered. In the proposed framework, VMC issues initial pseudonym to the vehicle in an encrypted channel. Therefore, the internal or insider attacker at CA, LTCA or PP cannot obtain the real identity of a vehicle. Similarly, after obtaining LTC, PP, and SPCs, the VMC is unaware of the valid identity of a vehicle during V2X communication. Furthermore, an external attacker cannot obtain any private information, because of encrypted and pseudonymized communication. All the communication in the proposed framework is encrypted and integrity protected, therefore, active and passive attacks are limited. Similarly, if the beacon contents are altered or a bogus message is inserted, the beacon signature cannot be authenticated.

Theorem A: The proposed framework is semantically protected against active and passive threats.

*Proof*: Let during the communication, an attacker gets an encrypted and pseudonymized message. In order to find the valid key, the attacker has to go through 2^128^(3.4x10^38^) keys. Where, the key size in the proposed framework is 128 bits. If there is a very powerful system with an attacker in the worst case that can compute 10^6^ decoding per microsecond. The total required time is (5.4x10^18^) years, which is impractical in ITS. It is extremely difficult for an attacker to eavesdrop the communication without the key. Further to enhance the proposed framework security, the nonce (*N*) is also used. Therefore, without the key and the nonce, it is impossible for an attacker to eavesdrop the communication. The proposed framework implements a distributed mechanism with strong security and privacy strategies.

Similarly, if an attacker tries to insert a bogus message or alter the contents of the message, the message signature cannot be authenticated and un-authenticated beacons are simply discarded. For an attacker that wants to launch active attacks, he/she needs in real time, the generation of key pairs. However, for keys generation, the attacker should have prior knowledge of the parameters as elaborated in Section III-D. Therefore, it is impractical to generate the keys that eliminate the active attacks concept. The ASPA implements strong privacy and security strategies among the vehicles and service providers that guarantee a high level of privacy.

Entropy is used to evaluate theorem *A*. Entropy elaborates the security of messages in a network. The discrete set of probabilities that can be expressed in case of ITS [14,56] is given below:

$$H(X)=-\sum_{i=1}^{\left|X\right|}p(x_{i})\log_{2}p(x_{i})=log_{2}|X|\;{if\;\forall }_{i}:p(x_{i})=\frac{1}{\left|X\right|},$$ (17)

and,

$$H_{max}=\log_{2}\left|N\right|.$$ (18)

The Shannon entropy further provides a technique to evaluate the probabilities, which measures the average minimum number of bits required to encrypt a text of symbols, based on its frequency in the text and is given by: *numBits = [H(X)]*. Where, *H(X)* represents the protected information. Highly secure communicated information can be represented through a high value of entropy. The high value of entropy ensures that passive and active attacks are impossible.

In ITS, information theory provides that for neighboring vehicles, the probabilities are as following:

Ω(x,y)={(x+1,y),(x−1,y),(x,y+1),(x,(y−1)}. (19)

In Eq (19), the coordinates of the vehicle are represented by *x* and *y*. The vehicle private key total weights corresponding probabilities are as following:

$$Z(x,y)=\sum_{\left(i,j)\varepsilon \Omega (x,y\right)}H(X)*W((x,y),(i,j)).$$ (20)

The key security, normal values at an iteration *t + 1* is represented by its neighboring normal values average weights at a previous iteration *t* and is given in Eq (21).

$$n^{t+1}(x,y)=\frac{\mu^{t+1}(x,y)}{{\left|\mu^{t+1}(x,y)\right|}^{2}},$$ (21)

where,

$$\mu^{t+1}(x,y)=\sum_{\left(i,j)\varepsilon \Omega (x,y\right)}n^{t}(i,j)\frac{W(\left(x,y),(i,j\right))}{Z(x,y)}.$$ (22)

The proposed framework security primitives guarantee a higher level of privacy i.e.:

$$d=\frac{H(X)}{H_{max}},$$ (23)

where *H(X)* shows the amount of secured information, *H*_*max*_ represents the maximum entropy, and *d* represents the level of security and privacy. For instance, if there are 50 vehicles and it is inferenced that there is an equal probability for all vehicles to be targeted, then $p(x_{i})=\frac{1}{50},\;p(x_{i})=0.02$, and the entropy is 5.64. Similarly, *H*_*max*_ = *Log*_2_|*N*| = 5.64, and *d* = 1. As discussed in Section III-E, *d* is a normalized quantity in the range of [0, 1]. ASPA framework guarantees a higher level of security and privacy for varying number of vehicles.

## IV. Revocation in ASPA

**Fig 6:**
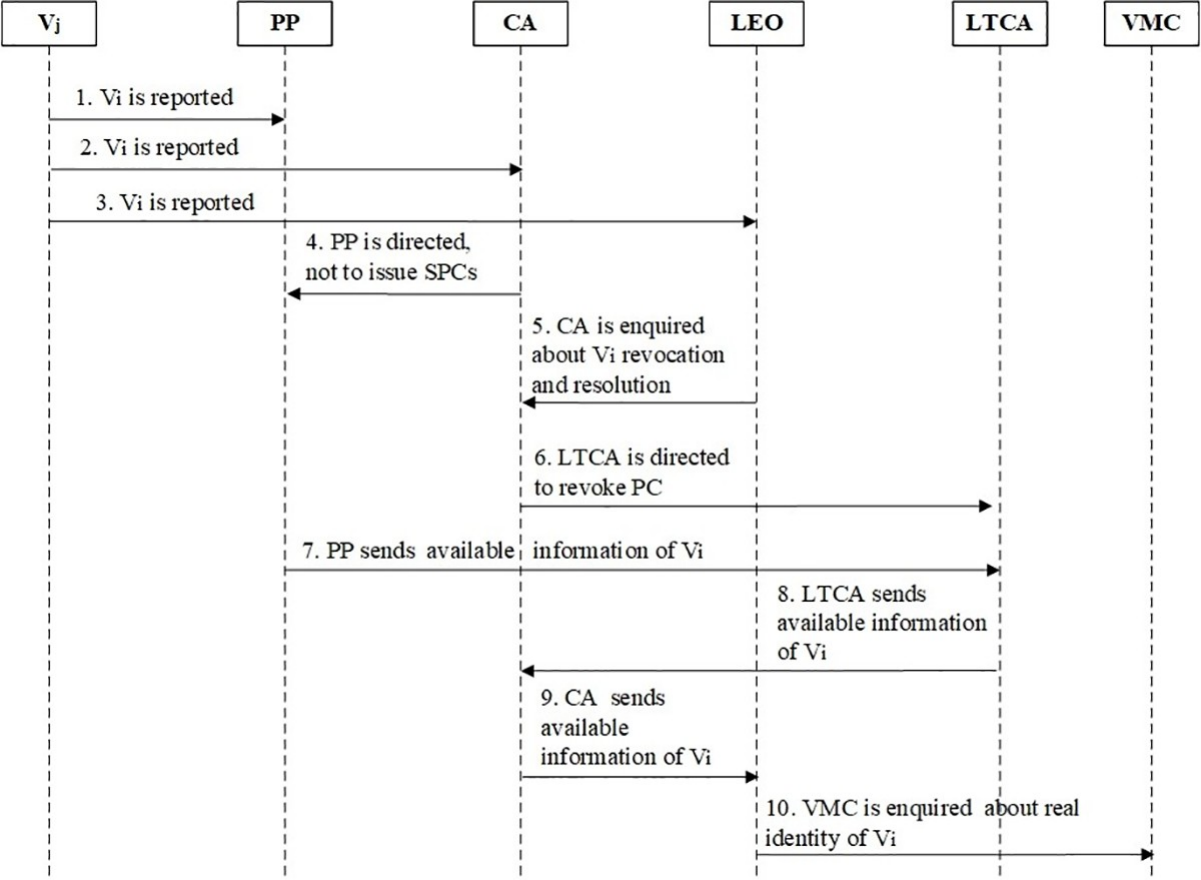
ASPA revocation and resolution process of malicious vehicle.

A malicious vehicle revocation and resolution process of the proposed ASPA framework is shown in Fig 6. Its steps are as follows:

- Step 1: The receiving vehicle of a bogus beacon (*V*_*j*_) that is affected, updates PP regarding the *V*_*i*_ (malicious vehicle). The SPCs are revoked and are broadcasted by the PP. The revoked broadcasted SPCs of *V*_*i*_ cannot be authenticated. Therefore, honest vehicles cannot be misguided.
- Step 2: The *V*_*j*_ updates CA for the revocation of *V*_*i*_.
- Step 3: The *V*_*j*_ updates LEO regarding *Vi* revocation from ITS and its accountability.
- Step 4: PP or cascaded PPs are informed by CA regarding not issue more SPCs and are directed to send the *V*_*i*_ pseudonymous information to LTCA.
- Step 5: CA is asked by LEO regarding *V*_*i*_ revocation from ITS and its real identity mapping.
- Step 6: The LTC is revoked by the CA after the LTCA replies. The LTCA is asked to revoke PC after PP replies and reports back regarding the pseudonym of *Vi*.
- Step 7: LTCA receives the pseudonym information of *Vi* from PP.
- Step 8: After receiving the PC of *Vi*, LTCA reports back to CA regarding *Vi* pseudonym.
- Step 9: LEO receives the pseudonym information from CA.
- Step 10: LEO forwards the pseudonym information of *V*_*i*_ to VMC for the mapping of its real identity.

In this mechanism, the *V*_*i*_ real identity can be disclosed. According to the laws of a particular country, the LEO takes action. The revocation and resolution protocol steps are presented in Algorithm 3.

Algorithm 3 ASPA revocation and resolution protocol

1: *V*_*j*_→*PP*:[(*Beacon message*)*K*_*Vi*_||*Pk*_*Vi*_||*SPC*]

2: *V*_*j*_→*CA*:[(*Beacon message*)*K*_*Vi*_||*Pk*_*Vi*_||*SPC||V*_*j*_*LTCpseudonym*]

3: *V*_*j*_→*LEO*:[(*Beacon message*)*K*_*Vi*_||*Pk*_*Vi*_||*SPC||V*_*j*_*LTCpseudonym*]

4: *CA*→*PP*:*Pk*_*PP*_[(*Beacon message*)*K*_*Vi*_||*Pk*_*Vi*_||*SPC*]

5: *LEO*→*CA*: [(*Beacon message*)*K*_*Vi*_||*Pk*_*Vi*_||*SPC||V*_*j*_*LTCpseudonym*]

6: *CA*→*LTCA*:*Pk*_*LTCA*_[(*Beacon message*)*K*_*Vi*_||*Pk*_*Vi*_||*SPC*]

7: *PP*→*LTCA*:*Pk*_*LTCA*_[*P*_3_]

8: *LTCA*→*CA*:*Pk*_*CA*_[*P*_2_]

9: *CA*→*LEO*:[*P*_1_]

10: *LEO*→*VMC*:[*P*_1_]

The beacons along with pseudonyms are kept in the vehicle OBU for a short time period. The beacons are authenticated quickly through pseudonyms and the public key. The vehicle (*V*_*i*_) signs the beacon through its private key, while the corresponding public key is linked with beacons, therefore, the communication cannot be refused. The signature verification process and the pseudonyms with beacons ensure the services of integrity and non-repudiation. Algorithm 4 shows the pseudo code of a malicious vehicle revocation and resolution process. In the proposed frame work of ASPA, the exponential growth of CRL is controlled through revocation of the most recent communication pseudonyms. Therefore, the revoked pseudonyms cannot be authenticated. Furthermore, a distributed/targeted attack cannot be carried out on a vehicle, as beacon consists of public key for the signature verification along with SPC. All the communication pseudonyms are provided through secure channels as discussed in Section III-F. Once a malicious vehicle or pseudonym is revoked, it cannot take part in the ITS. However, if *V*_*j*_ does issue a false positive claim, the LEO has its LTC pseudonym information. The LEO can take action against *V*_*j*_ because in this case, *V*_*j*_ is acting as a malicious vehicle. Therefore, LEO presents the LTC of *V*_*j*_ to CA and gets the pseudonym information of *V*_*j*_. The LEO enquires from VMC for the real identity of *V*_*j*_. The CA revokes LTC of *V*_*j*_, LTCA revokes the PC of *V*_*j*_, and PP is not required to issue more SPCs. In this way *V*_*j*_ can be revoked from the ITS network. According to the laws of a particular country, the LEO takes action.

Algorithm 4 Pseudo code of ASPA revocation and identity mapping

                      1: if *V*_*j*_ reports to LEO

2: if *V*_*j*_ reports to CA

3: if *V*_*j*_ reports to PP

4:        PP revokes the valid SPCs of *V*_*i*_

5:        LEO requests CA for mapping the factual identity of *V*_*i*_

6:        CA revokes LTC and LTCA revokes PC

7:        PP sends the available information of *V*_*i*_ to LTCA

8:        LTCA sends the available information to CA

9:        CA reports back to LEO regarding *V*_*i*_

10:     LEO requests VMC to reveal the original identity of *V*_*i*_

11:     end if

12: end if

13: end if

## V. Performance analysis

The proposed framework of ASPA is evaluated through Opportunistic Network Environment (ONE) simulator [7,57]. A core i7 laptop with 8GB RAM is used for the evaluation of the proposed framework. The experiments are performed 200 times. In order to perfectly evaluate the proposed framework, different speeds and network scenarios are considered. The parameters, which are considered in the simulations, are listed in Table 2. In order to analyze the performance of ASPA, the network parameters that are given below are analyzed.

- Average latency = Average (Message delivered time–Message created time)
- Overhead ratio = (Relayed messages–Delivered messages) / Delivered messages
- Delivery ratio = Delivered messages / Relayed messages

**Table 2:** ASPA design parameters.

Parameter name	Description
Duration	3600 seconds
Interface type	Simple broadcast interface IEEE 802.11P
Transmit speed	10 Mbps
Number of PP	1
Number of vehicles	5–100
Slow speed range	10 km/h to 50 km/h
Medium speed range	51 km/h to 80 km/h
High speed range	81 km/h to 120 km/h
Mobility model	Map based mobility
Routing protocol	Spray and Wait (SW)
Map of city	Helsinki
Transmit range	1000 meters
Area	10 km^2^

### A. Average latency

**Fig 7:**
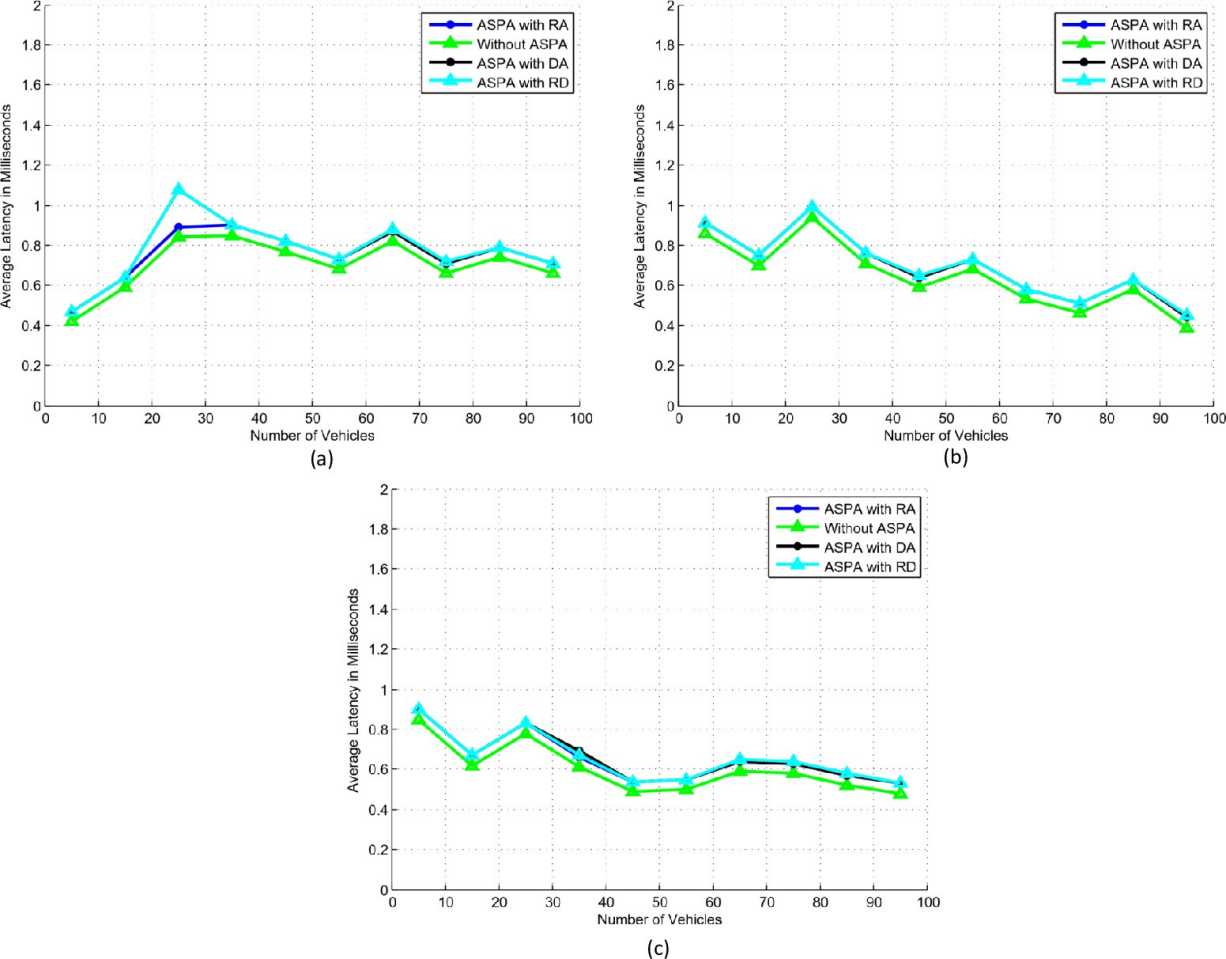
Average latency. (a) Slow speed. (b) Medium speed. (c) High speed.

The effect of average latency in different scenarios of sparse and dense networks with variable speeds of the proposed ASPA framework is shown in Fig 7. The results elaborate that without ASPA, ASPA with RA, ASPA with DA, and ASPA with RD network scenarios have no significant differences. In all forms of beacons, the same trend is observed. However, in Fig 7(A), the average latency increases. The reason for this increase is that vehicles with slow speed are advancing slowly and get congested. Therefore, more beacons have received that results to utilize more bandwidth. In all type of scenarios, less than one millisecond’s average latency is observed. Only in a sparse scenario of ASPA with RD, 1.1 milliseconds average latency is observed. Furthermore, in Fig 7(B) reduction in average latency is not smooth. The reason for this staircase is that vehicles with medium speeds are moving in the range of 51–80 km/h. Therefore, the distances among the vehicles are varying. Sometimes, due to less and more distances more or less beacons are received. In case of more beacons, more bandwidth is utilized. Similarly, in case of less beacons, less bandwidth is utilized.

In summary, implementation of the proposed framework in sparse network scenarios points to an increase in the average latency. While in dense network scenarios the average latency is either stable or reducing. The security and privacy layer does not affect communication.

### B. Overhead ratio

**Fig 8:**
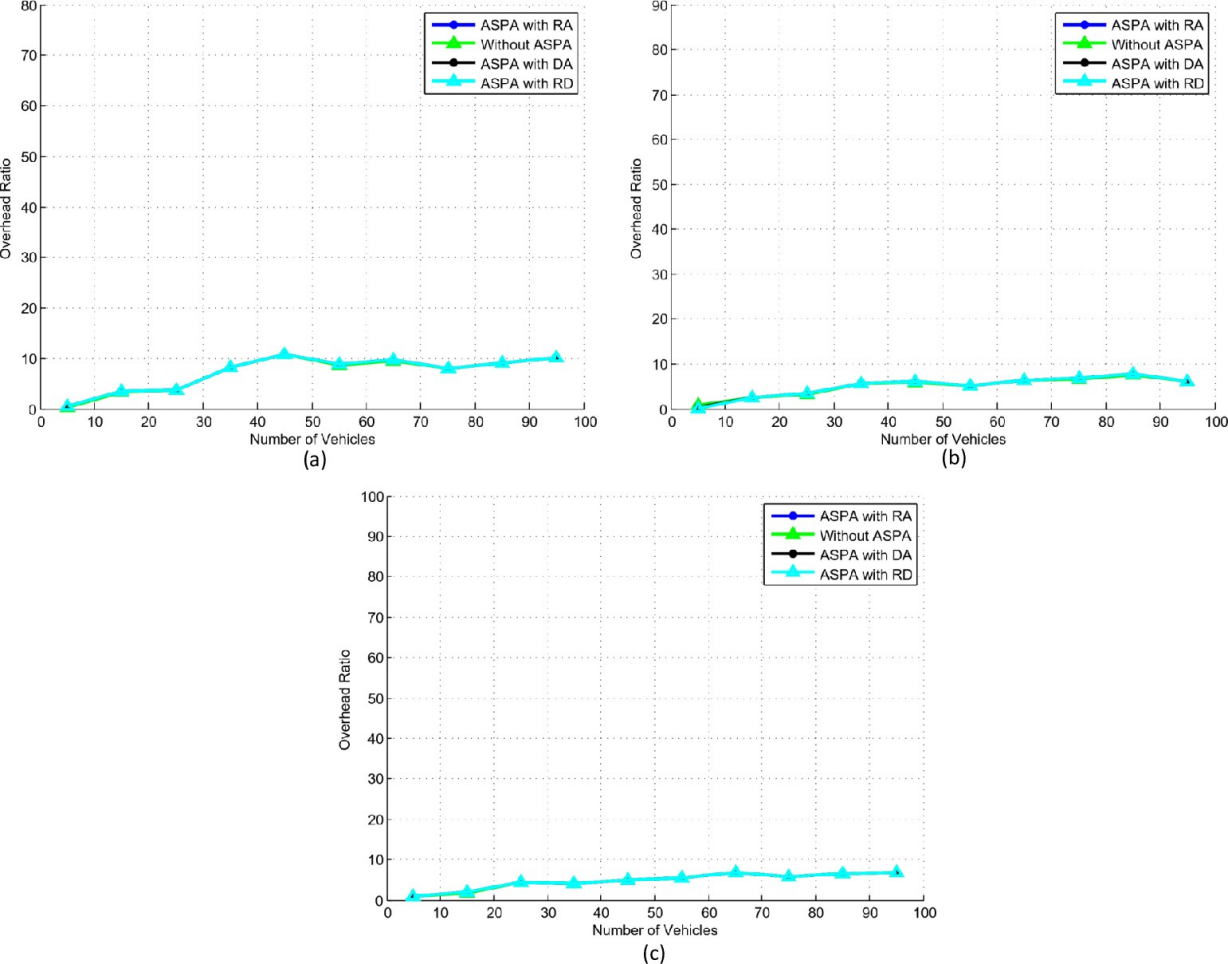
Overhead ratio. (a) Slow speed. (b) Medium speed. (c) High speed.

It is important to show the effect of overhead ratio/communication overhead with and without ASPA. The results retrieved during the simulations as shown in Fig 8 provide similar trends in all type of scenarios. A high overhead ratio is observed, when vehicles received more beacons. This is due to minimum distances among vehicles and more collisions. In all experiments, less than 2% communication overhead between ASPA and without ASPA is observed, which is negligible when considering security and privacy features.

### C. Delivery ratio

**Fig 9:**
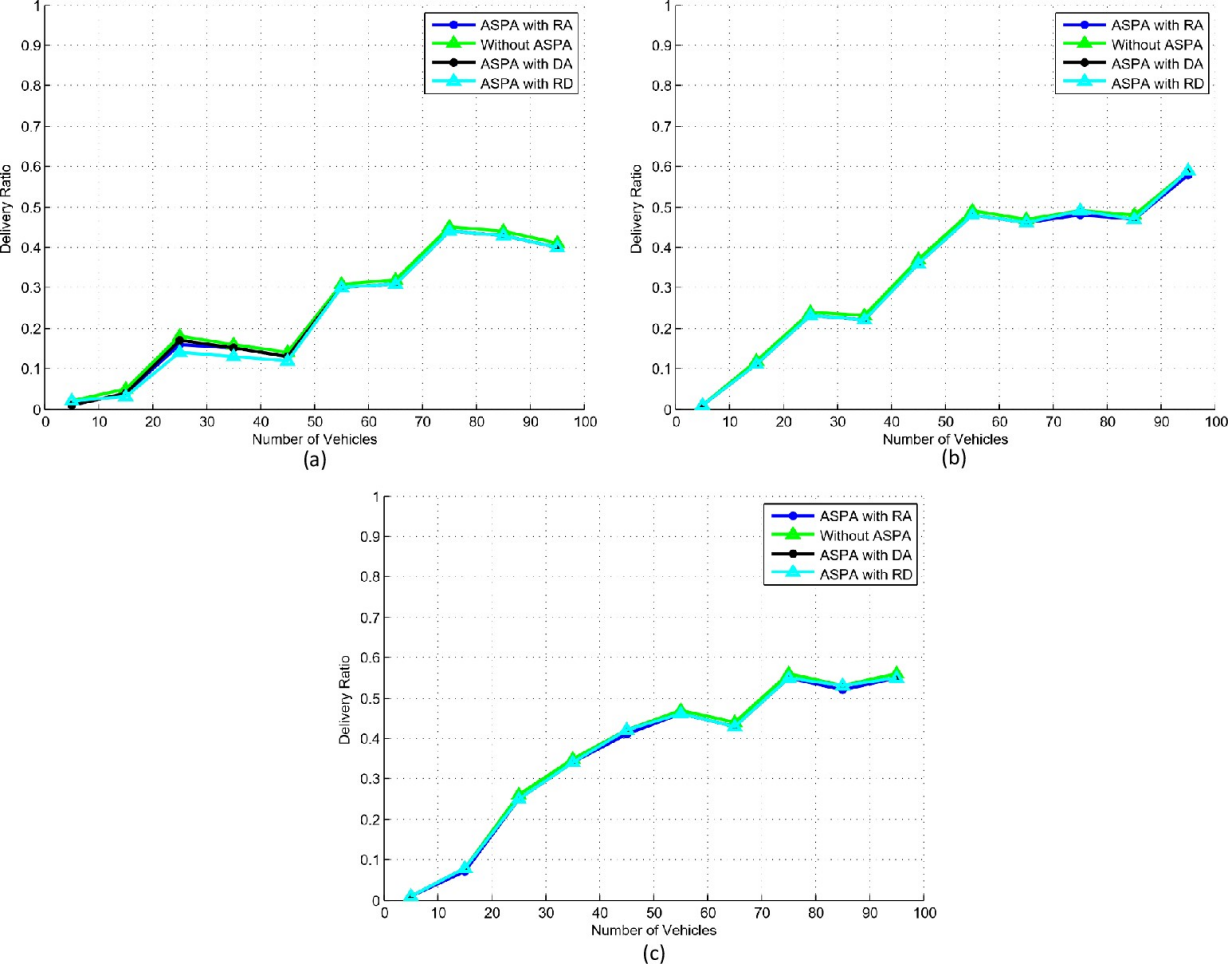
Delivery ratio. (a) Slow speed. (b) Medium speed. (c) High speed.

The delivery ratio is an important parameter that shows the appropriateness of the proposed ASPA framework. The results shown in Fig 9 follow no change in the status of delivery ratio with the implementation of ASPA. In medium and high speed scenarios, Fig 9(B) and 9(C), the delivery ratio either increases or remains stable. This is due to less bandwidth being occupied to accommodate moderate number of beacons, when there is an increase in the vehicles distances. While in Fig 9(A), the delivery ratio reduces after the number of vehicles goes beyond 75. The reason for this decrease is that the vehicles with slow speeds get closer and acquire more beacons. More bandwidth is required for more beacons and beacons are dropped. Therefore, the implementation of the security and privacy primitives in ASPA does not disturb the beacons delivery ratio.

### D. Computational cost analysis

The ASPA computational cost is evaluated and presented in Tables 3, 4 and 5, respectively. The beacon generation time is less than 4 milliseconds. Similarly, the beacon authentication time is less than one millisecond. Therefore, in the proposed framework of ASPA, vehicles efficiently generate and authenticate a large number of messages. In case of acquiring LTC and PC, a vehicle average time requirement is less than 4 milliseconds, respectively. Similarly, in the case of SPCs, the average time required is less than 5 milliseconds. Therefore, the efficient deployment of ASPA endorses service providers to efficiently process a large number of requests, simultaneously.

**Table 3:** Computational cost of ASPA with RA.

ASPA	Average computational time (ms)	Standard deviation (ms)
Message encryption	0.18	0.03
Signature generation	3.52	0.13
Message decryption	0.21	0.03
Signature verification	0.37	0.13

**Table 4:** Computational cost of ASPA with DA.

ASPA	Average computational time (ms)	Standard deviation (ms)
Message encryption	0.18	0.03
Signature generation	1.01	0.30
Message decryption	0.21	0.03
Signature verification	0.05	0.02

**Table 5:** Computational cost of ASPA with RD.

ASPA	Average computational time (ms)	Standard deviation (ms)
Message encryption	3.32	0.28
Signature generation	0.37	0.21
Message decryption	0.44	0.14
Signature verification	0.03	0.01

### E. Analysis of messages sizes

This subsection provides an analysis of the variously used security primitives in the process of pseudonyms generation and vehicle revocation. Table 6 shows the field sizes of the security primitives that are used in the proposed framework.

**Table 6:** ASPA individual field sizes.

Field name	Size in bytes
ID_VMC_	48
N	16
ID_V_	48
P_1_	16
K_V_	16
Sk_1_	16
TS_1_	5
LT_1_	5
ID_LTCA_	48
ID_PP_	48
Beacon message	34
Signature	34
Pk_Vi_	16
Pseudonym	16

During the registration phase of the ASPA framework, the sizes of messages between an ITS-S (vehicle) and the service providers are shown in Table 7. Similarly, during a malicious vehicle revocation and real identity tracing, the message sizes between the vehicle and the authorities are shown in Table 8.

**Table 7:** ASPA registration process messages sizes.

Steps	Size in bytes
Step 1	112
Step 2	144
Step 3	80
Step 4	80
Step 5	2
Step 6	180
Step 7	90
Step 8	154
Step 9	180
Step 10	90
Step 11	154
Step 12	74

**Table 8:** ASPA revocation and resolution process messages sizes.

Steps	Size in bytes
Step 1	100
Step 2	116
Step 3	116
Step 4	100
Step 5	116
Step 6	100
Step 7	16
Step 8	16
Step 9	16
Step 10	16

The results show that in all type of scenarios with security and privacy, there is no significant difference when compared with the scenarios of without security and privacy deployment. To further evaluate the behavior of ASPA suitability, the ASPA is implemented with different speeds in sparse and dense scenarios. No generous difference without security and privacy primitives and with ASPA is observed. This shows the real performance of the ASPA framework.

### F. Comparison with existing schemes

This subsection compares ASPA with the current PB and RSB/GSB approaches. In ASPA, the need for long communication pseudonyms pool and CRL large size is eliminated. A malicious vehicle, once revoked cannot be registered in the proposed framework. In addition, there is no need to keep a long pool of pseudonymous communication. In ASPA, it is ensured that if any of the servers are compromised, no useful information can be leaked. The criteria for high, medium, and low categorization is presented in Table 9, while ASPA is compared with existing security and privacy approaches in Table 10.

**Table 9:** Criteria for high, medium, and low categorization.

Parameters	High	Medium	Low
Computational cost	> 10 ms	5.1–10 ms	≤ 5 ms
Communication overhead	> 400 bytes	201–400 bytes	≤ 200 bytes
Storage requirements	> 1 MB	501–1024 KB	≤ 500 KB

**Table 10:** ASPA comparison with ITS existing security and privacy schemes.

Research paper	Computational cost	Communication Overhead	Storage requirements	Group management	Replay attack	Sybil attack	Side channel attack
[16]	High	High	High	No	Yes	Yes	No
[24]	High	High	High	No	No	Yes	No
[34]	High	High	High	No	Yes	Yes	Yes
[42]	High	High	High	No	Yes	Yes	Yes
[43]	Medium	High	High	No	No	Yes	Yes
[44]	Low	Medium	Low	Yes	Yes	Yes	Yes
[45]	Low	Medium	Low	No	Yes	Yes	Yes
[49]	Medium	High	Medium	Yes	Yes	No	Yes
[52]	Medium	Medium	Medium	Yes	No	Yes	No
[53]	High	High	High	Yes	Yes	Yes	No
ASPA	Low	Low	Low	No	No	No	No

The low computational costs and communication overheads of ASPA prove that it is an efficient and scalable framework. Furthermore, the security and privacy analysis is discussed in Section VI.

## VI. Security and privacy analysis

This section reviews the ASPA framework security and privacy services. Furthermore, different attack scenarios are examined.

### A. Security and privacy services

ASPA is a lightweight and trustworthy framework with restrictive obscurity. Due to the distributed mechanism, no single authority can know the vehicles real identities. The following security and privacy services are offered by the ASPA framework.

1. **Confidentiality and privacy:** The communication pseudonyms are acquired by vehicle through a secure channel. Therefore, the pseudonyms to pseudonym and pseudonym to real identity mapping are provided by the service authorities in a distributed and controlled way. No service authority can have access to the full mappings. Here, a hybrid approach of SKC and AKC are implemented for performance and security.
2. **Anonymity:** Controlled anonymity is used by vehicle through fictitious identities among vehicles and service providers. Vehicles real identities are preserved in a controlled manner.
3. **Integrity:** Trusted authorities, which are VMC, CA, LTCA, and PP control and monitor communication among vehicles. If any message/beacon is altered, the communication and signature cannot be confirmed.
4. **Authentication:** Anonymous authentication is achieved by verifying beacons, without revealing the real identity of source vehicles.
5. **Non-repudiation:** The trustworthy communication includes messages, signatures, and pseudonyms. The communication cannot be refused, once a vehicle is found awful. As the trusted authorities provided pseudonyms that are used in communication.

### B. Attack scenarios

Privacy and security in the ASPA framework is evaluated using the following attack scenarios:

1. Vehicles and authorities use encrypted communication. Therefore, the communication cannot be eavesdropped by attackers.
2. It is impractical for an adversary to obtain SPCs, without PC. Similarly, an attacker cannot obtain PC without LTC. It is also impossible for an attacker to get LTC without the endorsement of VMC.
3. In case, if a PP is attacked, no valuable information regarding the vehicles real identities can be leaked. As the PP maintains encrypted and pseudonymized information.
4. In case, if LTCA is attacked, no valuable information regarding the vehicles real identities can be leaked. As the LTCA maintains encrypted and pseudonymized information.
5. Similarly, in case, if CA is attacked, no useful information regarding the vehicles real identities can be leaked. The CA contains pseudonymized and encrypted information.
6. In ASPA, once a vehicle gets SPCs and if there is a successful attack on the VMC database. The attacker cannot collect any effective information about the vehicle real identity. As the vehicle is utilizing fictitious identities in the communication and the VMC database contains encrypted information.
7. Similarly, if an adversary attempts to inject a fake beacon or alter a beacon, the beacon signature cannot be authenticated.

The ASPA framework provides maximum privacy and restrictive anonymity because it is capable of handling all the above attacks.

## VII. Conclusion and future work

In ITS, due to intermittent connectivity and dynamic topology, security and privacy is a serious concern. In ASPA, multiple authorities are involved in pseudonyms generation to stay off articulation between pseudonyms and real identity mapping in an illegal way. Even in a malicious vehicle revocation phase, the real identity is preserved from the certificate authorities. ASPA can work efficiently in more complex scenarios and eliminate the concept of colluding attacks. The results present a stable increase in the delivery ratio. Similarly, in the results, overhead ratio and average latency are decreasing. ASPA with DA is one of the best approaches in terms of reduced computational overheads. In future, ASPA will be extended to work with multiple PPs and eventually it will be integrated with the cloud environment to form Internet of ITS-Ss.
